# Fatal Multi-Vessel Coronary Vasospasm: A Case Report

**DOI:** 10.7759/cureus.8271

**Published:** 2020-05-25

**Authors:** Hanane Aissaoui, Mohamed Boutaybi, Alla Ikbal, Noha Elouafi, Nabila Ismaili

**Affiliations:** 1 Cardiology, Mohammed I University/Mohammed VI University Hospital/Epidemiological Laboratory of Clinical Research and Public Health, Oujda, MAR; 2 Cardiology, Mohammed I University/Mohammed VI University Hospital, Oujda, MAR

**Keywords:** case report, coronary vasospasm, sudden death, acute coronary syndrome

## Abstract

We report a case of a 59-year-old female who experienced a history of an acute ST myocardial infarction. Percutaneous intervention and isosorbide dinitrate perfusion were successful in reversing the severe vasospasm on the left anterior descending, the first diagonal, and posterior interventricular arteries. The patient received calcium channel blockers and nitrates with a good in-hospital clinical evolution. One month later, the patient presented to the ED with chest pain leading to cardiac arrest despite cardiopulmonary resuscitation. This case highlights the fatal outcome of coronary artery vasospasm.

## Introduction

Although it is a rare condition, the coronary artery vasospasm angina is an underestimated cause of cardiac arrest [1]. Recognizing its etiology is difficult. Patients are often young females with fewer risk factors presenting with different noncardiac symptoms which may underestimate the cardiac cause of chest pain and lead unfortunately in some cases, to sudden death, often out of hospital [2]. The management of this serious condition includes calcium channel blockers and nitrates which have been known to be effective in preventing coronary vasospasm, but in some cases, life-threatening ventricular arrhythmias may occur despite optimal medical treatment [3]. Defibrillator implantation may help in those cases to prevent sudden cardiac events. In this article, we describe a rapid and unfavorable evolution of a 59-year-old female who experienced fatal coronary vasospasm.

## Case presentation

**Figure 1:**
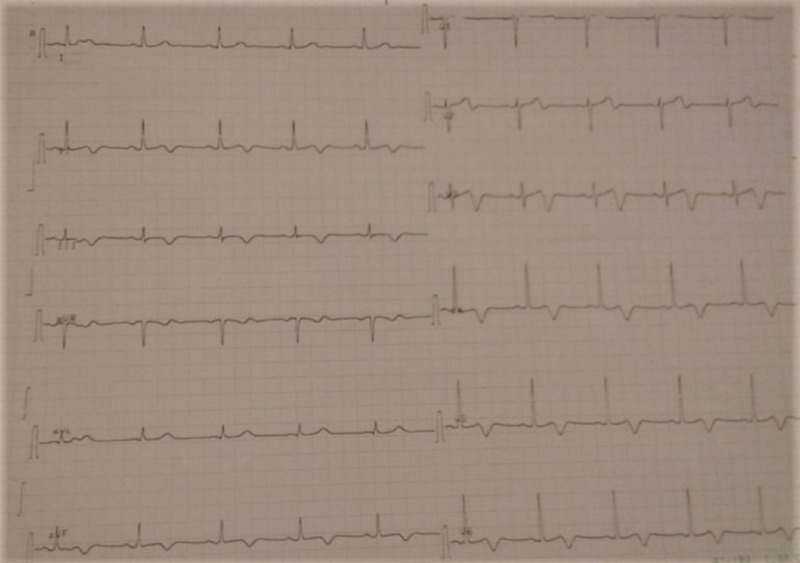
T negative waves on the inferior and the anterior leads on EKG. EKG, electrocardiogram

**Figure 2:**
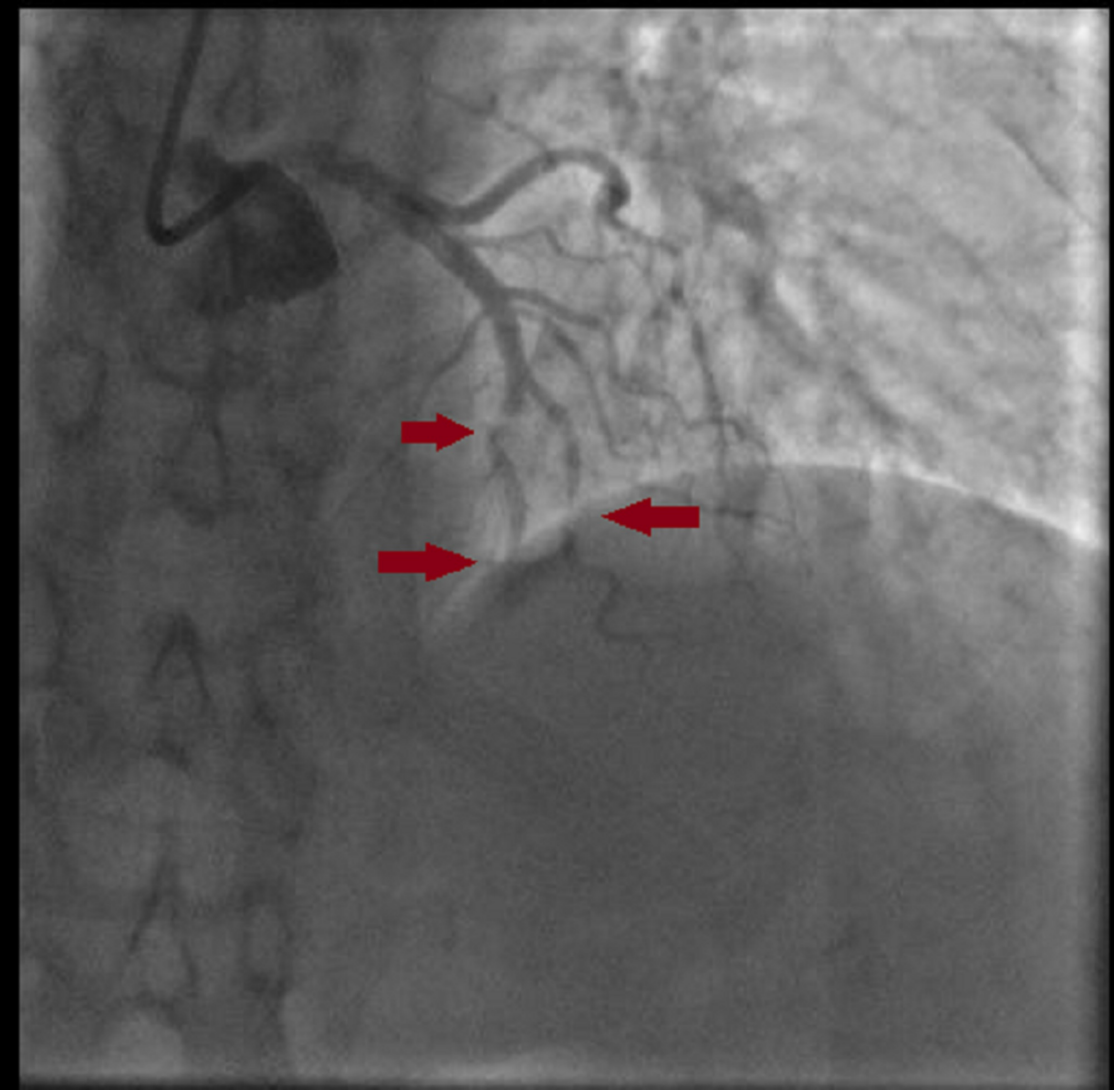
A diffuse vasospasm in the LAD and the diagonal artery. LAD, left anterior descending artery

**Figure 3:**
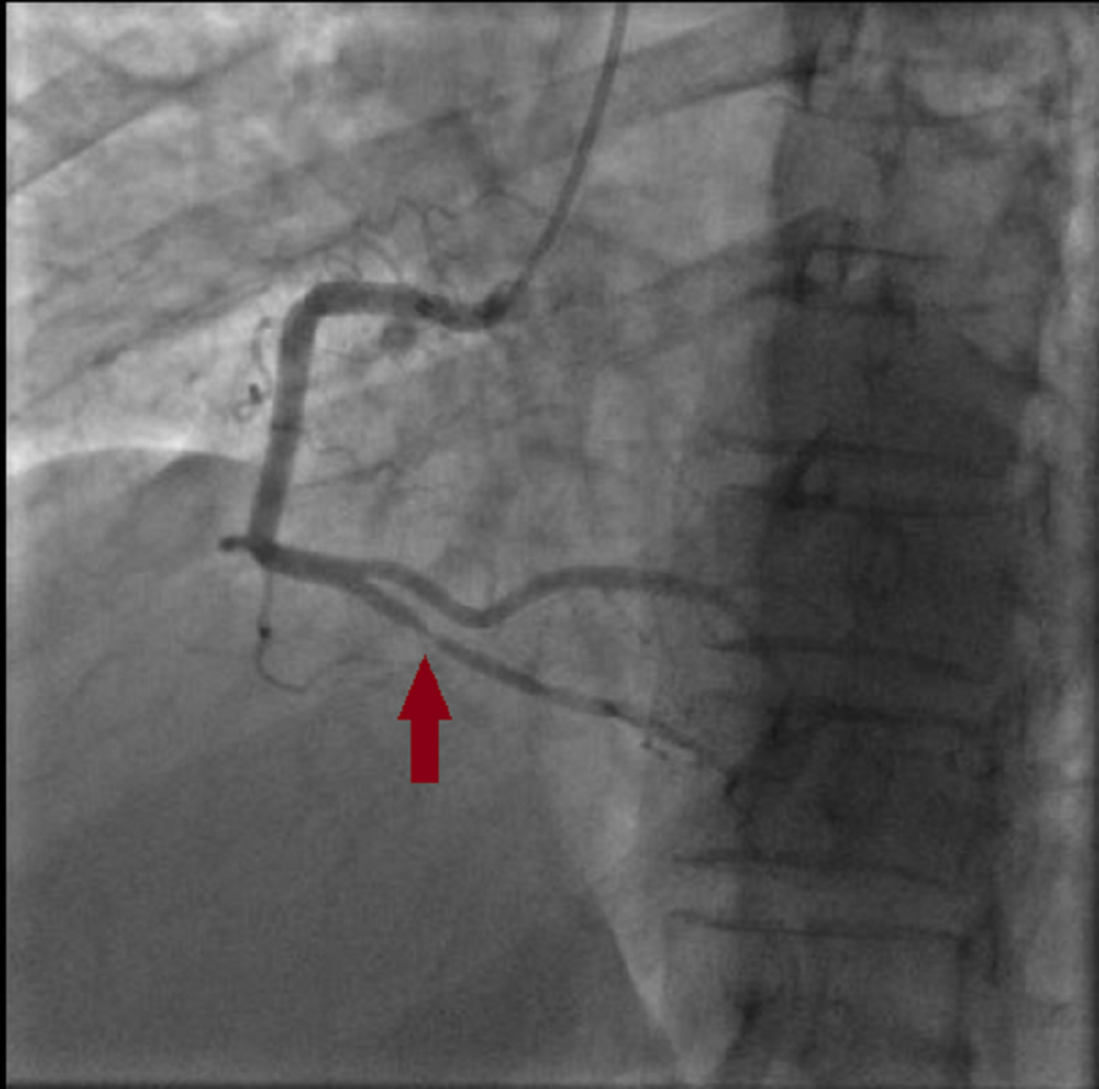
Vasospasm in the posterior interventricular artery.

A 59-year-old female with a past medical history of hyperlipidemia and chronic obstructive pulmonary disease presented to our department with a one-month history of intermittent angina. Her symptoms worsened three days before her admission, and she complained of an intense durable chest pain radiating to her jaw; her electrocardiogram (EKG) showed a normal sinus rhythm with 68 beats per minute (bpm), and negative T-waves at the inferior and the anterior leads (Figure 1) with increased troponin levels (from 614 to 4725 ng/L). A coronary angiogram was performed and showed multifocal vasospasm of the left anterior descending artery (LAD), the diagonal artery (Figure 2), and the posterior interventricular artery (Figure 3) with a good response to intra-cardiac isosorbide dinitrate. 

**Figure 4:**
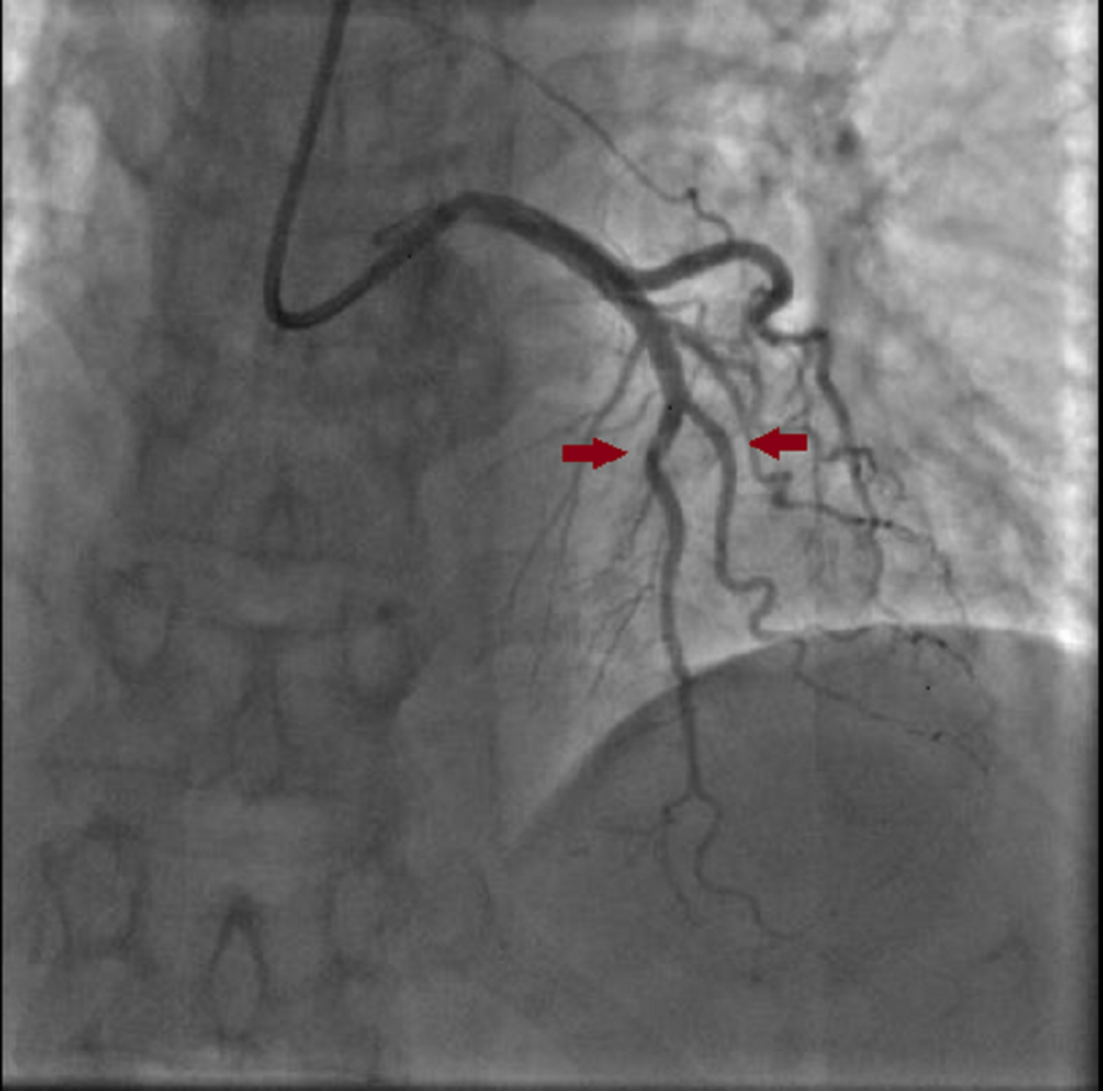
Coronary angiogram after isosorbide dinitrate injection showing a total relief of the vasospasm.

**Figure 5:**
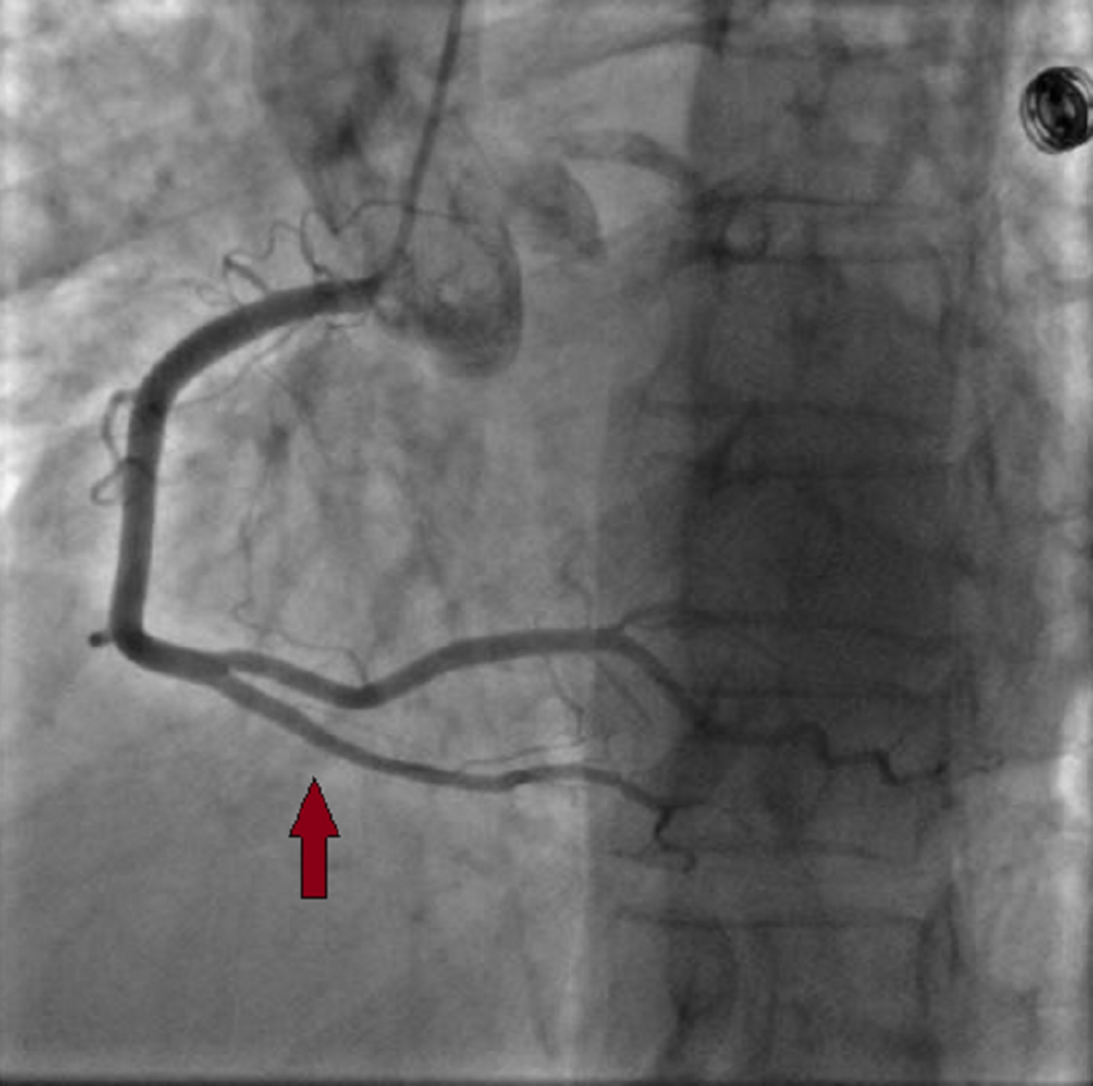
Relief of the vasospasm in the posterior interventricular artery after isosorbide dinitrate perfusion.

One day after the intervention, the patient suffered from a recurrence of chest pain associated with an ST-elevation in the apico-lateral and inferior leads. We treated the patient with a perfusion of isosorbide dinitrate (2 mg) before the cardiac catheterization. The coronary angiogram revealed normal coronary arteries with no spasm image under isosorbide dinitrate perfusion (Figures 4-5).

Postcatheterization treatment of the patient was started with aspirin 75 mg/day, diltiazem 120 mg/day, isosorbide mononitrate 20 mg*3/day, and trinitrate spray inhalation 400-800 µg/day with an improved decrease of cardiac enzymes. Transthoracic ultrasound showed a left ventricular ejection fraction of 57% with akinesis of the apical segment of the anterior, anteroseptal walls. The patient remained stable under surveillance in the intensive care cardiology unit without the recurrence of angina. The EKG surveillance did not show any conductive or ventricular arrhythmia. The evolution was favorable under medical treatment with no recurrence of chest pain. One month after her discharge, the patient reported chest pain with the same previous clinical characteristics. Although previously effective, nitrate and calcium channel blockers treatment did not relieve her pain, which encouraged her to consult the ED. She was admitted to the ICU in a cardiac arrest (asystole) and died after 45 minutes of cardiopulmonary resuscitation. No specific cause was found for her cardiac arrest except the known vasospasm. 

## Discussion

Coronary artery spasm (CAS) is characterized by recurrent episodes of angina at rest associated with transient ST-segment elevation on the EKG [4]. It is caused by focal vasospasm of one or more coronary arteries, usually within a normal vessel, due to the vasoconstriction of the epicardial coronary arteries. Consequently, this leads to myocardial ischemia associated with chest pain, unstable angina, acute coronary syndrome, and in some cases, a sudden death [5]. The prevalence of coronary vasospasm is higher in female Caucasians, with a history of smoking, alcohol drinking, migraine, or under antidepressant treatment. It is also frequent in patients with lipid metabolism disorders, diabetes as well as abnormal or hyper-contractility of vascular smooth muscles. Magnesium deficiency and multiple genetic polymorphisms have also been found to be linked to the physiopathological mechanisms of CAS [5-6]. Our patient was a young female patient with dyslipidemia without any other risk factors. Usually, CAS does not always present with typical chest angina. EKG could also be normal and lead the physician to exclude cardiac causes [6]. A cohort study found that sudden cardiac arrest is associated with age, hypertension, hyperlipidemia, family history of sudden cardiac death, multivessel spasm, and LAD spasm [7]. Our patient had hyperlipidemia and a multivessel spasm with a diffuse LAD spasm. Lee et al. showed that early repolarization patterns of horizontal/descending ST-segment elevation and right-sided coronary artery vasospasms are associated with recurrent sudden cardiac death events [3]. Usually, the vasospasm appears in the LAD and right coronary artery (RCA) rather than the left circumflex coronary artery (LCX) [5]. Provocation tests of coronary artery vasospasm are helpful for the diagnosis of vasospastic angina. However, these tests have a potential risk of cardiac arrhythmias. The provocative coronary vasospasm testing implies the administration of a provocative drug usually the acetylcholine and ergonovine during coronary angiography while monitoring the patient’s symptoms [3, 8]. In our case, we were successful in observing the fading of the spasm during the coronary angiogram. The spasm has vanished once we injected intracardiac isosorbide dinitrate. Nonspecific vasodilators such as nitrates and calcium channel blockers are considered as the standard treatment and may improve the clinical outcomes of patients with vasospastic angina [3]. Percutaneous coronary stenting can be beneficial for patients who present refractory symptoms to optimal medical treatment and have focal vasospasm [9]. This was impossible for our patient case, who had multi-vessel vasospasms. Patients that are refractory to pharmacologic and percutaneous invasive therapy present most likely a severe vasomotor disorder and need more intensive long medical treatments and continued surveillance [10-12]. In some cases, despite good outcomes under medical treatment, 6.1% of patients suffer from ventricular arrhythmia events in the long outcome which requires implantable cardioverter-defibrillator (ICD) use as a considerable tool to improve the long-term prognosis [13]. However, the clinical studies on the effectiveness of the ICD are controversial but its role is undeniable to prevent sudden death due to malignant ventricular arrhythmias [14]. Ishihara et al. showed that using optimal medical treatment with an ICD may reduce the recurrence of ventricular arrhythmia for some patients with coronary vasospasm who have been successfully resuscitated from ventricular fibrillation or cardiac arrest [15]. On the other hand, Ahn et al. found that there was no significant trend of cardiac deaths in patients with ICDs [7]. Coronary vasospasm is an underestimated cause of out hospital cardiac arrest especially within young patients [16]. Krahn et al. reported that 11% of patients experienced cardiac arrest due to vasospasm. Moreover, a postmortem autopsy study showed similar findings with 12% of patients that were found to have coronary vasospasm as a cause of cardiac arrest [17-18]. The prognosis of coronary vasospasm is related to the number of the affected arteries: a diffuse spasm has a poorer prognosis than single-vessel involvement [18].

## Conclusions

Coronary spasm is a rare entity that may cause sudden death in young patients even without a history of heart disease. Our case highlights the necessity to use multiple vasodilator treatment to prevent the likelihood of future episodes of coronary vasospasm. Notably, the insertion of ICD in high-risk patients to prevent arrhythmia and sudden death is recommended.

## References

[1] Naqvi SY, Hanley A, Crowley J (2014). Ventricular fibrillation due to coronary vasospasm. BMJ Case Rep.

[2] Beijk MA, Vlastra WV, Delewi R (2019). Myocardial infarction with non-obstructive coronary arteries: a focus on vasospastic angina. Neth Heart J.

[3] Lee KH, Park HW, Cho JG (2014). Predictors of recurrent sudden cardiac death in patients associated with coronary vasospasm. Int J Cardiol.

[4] Meller J, Pichard A, Dack S (1976). Coronary arterial spasm in Prinzmetal's angina: a proved hypothesis. Am J Cardiol.

[5] Teragawa H, Oshita C, Ueda T (2018). Coronary spasm: It's common, but it's still unsolved. World J Cardiol.

[6] Picard F, Sayah N, Spagnoli V, Adjedj J, Varenne O (2019). Vasospastic angina: a literature review of current evidence. Arch Cardiovasc Dis.

[7] Ahn JM, Lee KH, Yoo SY (2016). Prognosis of variant angina manifesting as aborted sudden cardiac death. J Am Coll Cardiol.

[8] Takagi Y, Yasuda S, Takahashi J (2013). Clinical implications of provocation tests for coronary artery spasm: safety, arrhythmic complications, and prognostic impact: multicentre registry study of the Japanese Coronary Spasm Association. Eur Heart J.

[9] Song JK (2018). Coronary artery vasospasm. Korean Circ J.

[10] Tanabe Y, Itoh E, Suzuki K (2002). Limited role of coronary angioplasty and stenting in coronary spastic angina with organic stenosis. J Am Coll Cardiol.

[11] Martí V, Ligero C, García J, Kastanis P, Guindo J, Domínguez de Rozas JM (2006). Stent implantation in variant angina refractory to medical treatment. Clin Cardiol.

[12] Chu G, Zhang G, Zhang Z, Liu S, Wen Q, Sun B (2013). Clinical outcome of coronary stenting in patients with variant angina refractory to medical treatment: a consecutive single-center analysis. Med Princ Pract.

[13] Rodríguez-Mañero M, Oloriz T, le Polain de Waroux JB (2018). Long-term prognosis of patients with life-threatening ventricular arrhythmias induced by coronary artery spasm. Europace.

[14] Sun J, Feng L, Li F, Zhang Y, Dong J (2017). An interesting implantable cardioverter defibrillator treatment for lethal ventricular arrhythmias caused by coronary artery spasm: a case report. Medicine (Baltimore).

[15] Ishihara A, Tanaka T, Otsu Y (2012). Prognosis of patients with coronary vasospasm after successful resuscitation from ventricular fibrillation. J Arrhythm.

[16] Kobayashi N, Hata N, Shimura T (2013). Characteristics of patients with cardiac arrest caused by coronary vasospasm. Circ J.

[17] Krahn AD, Healey JS, Chauhan V (2010). Systematic assessment of patients with unexplained cardiac arrest: Cardiac Arrest Survivors With Preserved Ejection Fraction Registry (CASPER). Circulation.

[18] Hill SF, Sheppard MN (2010). Non-atherosclerotic coronary artery disease associated with sudden cardiac death. Heart.

